# Acaricidal activity of plant-derived essential oil components against *Psoroptes ovis in vitro* and *in vivo*

**DOI:** 10.1186/s13071-019-3654-x

**Published:** 2019-08-29

**Authors:** Zhenzhen Chen, Wouter van Mol, Marieke Vanhecke, Luc Duchateau, Edwin Claerebout

**Affiliations:** 1 Laboratory of Parasitology, Faculty of Veterinary Medicine, Ghent University, Salisburylaan 133, 9820 Merelbeke, Belgium; 2 Biometrics Research Center, Faculty of Veterinary Medicine, Ghent University, Salisburylaan 133, 9820 Merelbeke, Belgium

**Keywords:** *Psoroptes ovis*, Cattle, Acaricide, Essential oils, *In vitro*, *In vivo*

## Abstract

**Background:**

Treatment of *Psoroptes ovis* in cattle is limited to topical acaricides or systemic treatment with macrocyclic lactones. Treatment failure of macrocyclic lactones has been reported. The aim of this study was to evaluate a potential alternative treatment against *P. ovis*.

**Methods:**

The acaricidal activity against *P. ovis* of four plant-derived essential oil components, i.e. geraniol, eugenol, 1,8-cineol and carvacrol, was assessed *in vitro* and *in vivo*. *In vitro* contact, fumigation and residual bioassays were performed. In addition, 12 Belgium Blue cattle were artificially infested and treated topically once a week for three successive weeks with carvacrol in Tween-80 (treatment group) or with Tween-80 alone (control). The efficacy of carvacrol was determined by the reduction in lesion size and mite counts. Six additional animals were topically treated with carvacrol to assess local adverse reactions.

**Results:**

Three components showed a concentration-dependent acaricidal activity in a contact assay, with LC_50_ of 0.56, 0.38 and 0.26% at 24 h for geraniol, eugenol, and carvacrol, respectively. However, 1,8-cineol showed no activity at any of the tested concentrations in a contact bioassay. In a fumigation bioassay, carvacrol killed all mites within 50 min after treatment, whereas geraniol, eugenol and 1,8-cineol needed 90 to 150 min. Following a 72 h incubation period in a residual bioassay, carvacrol killed all mites after 4 h of exposure to LC_90,_ while geraniol and eugenol killed all mites only after 8 h exposure. Based on these results, carvacrol was further assessed *in vivo*. Mite counts in the treatment group were reduced by 98.5 ± 2.4% at 6 weeks post-treatment, while in the control group the mite population had increased. Topical application of carvacrol only caused mild and transient erythema 20 min after treatment. No other side effects were observed.

**Conclusions:**

Considering the strong acaricidal activity of carvacrol *in vitro* and *in vivo* and the mild and transient local side effects, carvacrol shows potential as an acaricidal agent in the treatment of *P. ovis* in cattle.

## Background

*Psoroptes ovis* is a common ectoparasitic mite of sheep, cattle and rabbits. It is the causative agent of a ubiquitous skin disease, which is commonly referred to as psoroptic mange. Psoroptic mange affects animal health and can ultimately have a lethal outcome, leading to financial losses and animal welfare issues [1–5].

Psoroptic mange can be treated by local administration of amitraz or pyrethroids or systemic administration of macrocyclic lactones. Although chemical drugs have been highly effective against psoroptic mange in the past decades [6–9], their frequent and/or incorrect usage has resulted in a reduced efficacy due to emerging drug resistance, leading to inadequate control [10–12]. Consequently, there is an urgent need to develop new effective and safe acaricidal agents for treatment and control of animal acariasis.

Several studies in recent years have focused on the bioactive effects of plant-derived products against ectoparasites, including essential oils. For instance, some essential oils, such as oregano oil, lavender, tea tree oil and neem oil, have been tested against *Sarcoptes scabiei* and *Psoroptes* spp. and seem to have strong acaricidal efficacy *in vitro* [13–20]. Several essential oils were also tested *in vivo* against *S. scabiei* in rabbits, goats and pigs [21–24], against *P. cuniculi* in rabbits [13, 25–27] and against *Chorioptes texanus* in cattle [28]. Essential oils are volatile oils which are naturally produced by plants as secondary compounds, and are commercially available as concentrated products containing volatile aroma compounds [29, 30]. In general, essential oils are particularly attractive over chemical acaricides, given their low animal toxicity and short environmental persistence [31], although a limited number of studies have displayed phytotoxic effects of essential oils [32]. Furthermore, essential oils are known to have a complex chemistry which significantly hampers the development of drug resistance against these compounds. On the downside however, essential oils often represent a complex mixture of compounds which makes it difficult to pinpoint the acaricidal activity to a certain compound or a composition of compounds. Another potential disadvantage is skin irritation, which has been described in humans [33].

In previous research, geraniol, eugenol, carvacrol and 1,8-cineol showed good acaricidal activity against *S. scabiei* [17, 34], *P. cuniculi* [35–37] or *P. ovis* [38] *in vitro*, but to our knowledge pure compounds have not been tested *in vivo*. In the present study, we aimed to assess the potential acaricidal activity of four essential oil compounds (geraniol, eugenol, carvacrol and 1,8-cineol) against *P. ovis in vitro* and *in vivo* in cattle, to develop novel and safe antiparasitic drugs.

## Methods

### Compounds

Geraniol (NO: 48798), eugenol (NO: 35995), carvacrol (NO: 42632), 1,8-cineol (NO: 29210), Tween-80 (NO: P1754), paraffin oil (NO: 18512) and mineral oil (NO: M5904) were purchased from Sigma-Aldrich (Brussels, Belgium) for *in vitro* assays. Carvacrol (NO: W224511) was purchased from Sigma-Aldrich for an *in vivo* assay. All compounds were commercially available as samples purified to 99% (analytical reagent > 99%).

### *Psoroptes ovis* mites

Adult *P. ovis* mites were isolated from naturally infested cattle. Skin scrapings were collected from the edge of the skin lesions on the back and were stored at 10 °C overnight. The next day, collected skin scrapings were incubated in Petri dishes at 30 °C for 10 min. Live adult mites (male and female) were picked up with a needle and identified under a stereomicroscope (400× magnification) as *P. ovis* based on morphological criteria [39].

### Contact assay

In order to evaluate the acaricidal activity of four plant-derived essential oil compounds (geraniol, eugenol, carvacrol and 1,8-cineol) on adult *P. ovis* mites, an acaricide contact assay (immersion test) was performed as described by Fichi et al. [26]. Two-fold serial dilutions of the four compounds were made in paraffin oil (geraniol, carvacrol and 1,8-cineol) or in mineral oil (eugenol) to reach final concentrations of 5.0, 2.5, 1.25, 0.63, 0.32 and 0.16 %. Next, 10 adult mites were immersed in each of the different oil concentrations and inspected under a stereomicroscope at 1, 2, 4, 6, 8, 10, 12 and 24 h post-immersion. Liquid paraffin oil or mineral oil was used as a negative control. Immobility of adult mites and a lack of reactions or persistent immobility within 1 min following stimulation with a needle were considered indications of death. The immersion test for all dilutions was performed at 30 ± 1 °C and at 55 ± 5% relative humidity. The test was repeated twice independently and three replicates were run concurrently for all tested dilutions.

### Fumigation assay

To assess the fumigant toxicity of the four compounds, a fumigation assay was performed as described by Fang et al. [14]. A droplet (15 μl) of the undiluted compound was added to the bottom of a plastic Petri dish (3 cm in diameter). Ten adult mites were placed on the centre of the lid and covered with a filter paper to restrain the mites. The Petri dish was subsequently closed. Control Petri dishes were treated with paraffin oil or mineral oil. The mites were observed every 10 min under a stereomicroscope until 150 min after the start of the experiment. At each timepoint, the number of viable mites was counted. All tests were performed at 30 ± 1 °C and at 55 ± 5% relative humidity. The test was performed twice independently and three replicates were run in each test.

### Residual assay

LC_50_ and LC_90_ values obtained from the contact assays were used in a residual activity assay to determine the residual activity of geraniol, eugenol and carvacrol. The effective concentrations (LC_50_ and LC_90_) of these compounds were prepared in paraffin oil or mineral oil in Eppendorf tubes (1.5 ml) and incubated with the lids open for 24, 48, 72, 120 and 168 h. Following incubation, batches of 10 adult mites were brought into contact with the different compounds as described above for the immersion test. Paraffin oil or mineral oil was used as a negative control. Live mites were checked at 1, 4, 8 and 24 h post-treatment. All tests were performed twice at 30 ± 1 °C and at 55 ± 5% relative humidity and three replicates were run in each test for all tested compounds.

### *In vivo* assay

Twelve healthy Belgian Blue calves (4–12 months-old) were included in the *in vivo* study. All calves were free of mite and lice infestation as determined by thorough body inspection and examination of skin scrapings prior to the experiment. The animals were randomly allocated to either the control group or the treatment group. To assess the acaricidal activity of carvacrol *in vivo*, calves were experimentally infested with 400 *P. ovis* mites (nymphs and adults) by placing mites directly onto the skin at the left and right side of the withers (200 mites/side) with a filter paper to prevent them from escaping. The filter papers were kept in place by tying a rubber band around the animal’s chest. All animals were individually stanchioned and refrained from scratching by a metal frame around the neck throughout the trial to limit self-grooming behaviour and prevent disruption of the developing lesions [6, 7].

In the treatment group, calves received a topical treatment with 2% carvacrol emulsified in Tween-80 (2%) [24], while in the control group calves received treatment with Tween-80 (2%) only. In both groups, approximately 0.4 ml of the emulsion was applied per cm^2^ of lesion [15] by using a 500 ml plastic garden sprayer which was adjusted to give a steady jet (as opposed to a fine spray). The sprayer was calibrated prior to use; a single squirt was estimated to deliver approximate 0.5 ml. In addition, as a cross check, the volume of compound solution remaining at the end of the treatment application was measured. The treatment was applied three times with weekly intervals. Following treatment administration, the lesion sizes were monitored weekly starting from the day of infestation (Week 0) until the end of the study (Week 12). A clinical index (CI) was assigned to all animals by recording the skin lesions (on both sides of the animal) on a silhouette [4]. At each visit, the investigator assessed the surface of all the lesions on the animal’s body in the grid and then counted the percentage affected by lesions. CI was recorded as follows: 0, healthy skin; 1, lesions surface < 10% of body surface; 2, lesions surface 10–20% of body surface; 3, lesions surface 20–30% of body surface; 4, lesions surface > 30% of body surface.

Additionally, the number of *P. ovis* mites in active lesions was counted before treatment (Week 6 post-infestation) and weekly after receiving the third treatment (Week 9–12). This was done by taking skin scrapings from the edges of active lesions or, if lesions regressed during the study, from the area where active lesions were at the study commencement, according to the guidelines of the World Association for the Advancement of Veterinary Parasitology [40]. In total three lesion sites per animal were sampled by scraping an area of 9 cm^2^ per lesion. Samples were examined within 24 h of collection to identify and count *P. ovis* mites.

The acaricide efficacy of carvacrol was determined by comparing the reduction in lesion size (CI) and the number of viable mites in the treated group with the control group using Abbott’s formula [% efficacy = (C − T)/C × 100] [41]. In this formula, C and T are the geometric mean lesion size or mite count in the control group and treated group, respectively.

On Week 12, all animals were treated topically with amitraz (Taktic^®^) at the recommended dose. Amitraz treatment was applied two times with a one-week interval. Animals were handled and treated with due regard for their welfare and in compliance with animal welfare legislation.

### Skin irritation assay

Six Belgian Blue calves (4–12 months-old) were included. Animals were handled and treated with due regard for their welfare and in compliance with animal welfare legislation. The test area (15 × 15 cm) at both thighs of all animals was shorn on Day − 2. Two days later (Day 0), the test area was washed with water and the animals were allowed to dry. The treatment side and the control side were then treated once topically with 2% carvacrol in Tween-80 (2%) emulsion or only Tween-80 (2%), respectively, at a rate of approximately 0.4 ml/cm^2^ of skin. Following treatment, locations of erythema and oedema were monitored at 20 min, 6 h, 24 h and 48 h post-treatment. Skin was evaluated for erythema and oedema according to the Draize system [42] for erythema (0, no change; 1, very slight change; 2, pale red in defined area; 3, definite red in well-defined area; 4, crimson red) and for edema (0, no change; 1, very slight change; 2, slight change with edges barely defined; 3, moderate change with area raised 1 mm; 4, severe change with area raised > 1 mm and extending beyond the exposure area).

To assess histological alterations during the study, skin biopsies were removed from each animal before treatment and at 20 min, 6 h, 24 h and 48 h post-treatment using a disposable 4-mm biopsy punch (Kai Europe GmbH, Solingen, Germany), following the administration of a local anaesthetic (3 ml, 4% procainii hydrochloridum and 0.0036% adrenalini tartras) (KELA, Sint-Niklaas, Belgium). Skin tissues were fixed in 4% paraformaldehyde, processed and embedded in paraffin wax. Subsequently 5-μm sections of tissue were stained with haematoxylin and eosin (H&E) or Giemsa and examined microscopically. The width of the *stratum corneum* and eosinophil and mast cell counts were used to describe the histopathological changes of the skin sections.

### Statistical analysis

Survival times of mites in the treatment and control groups were analyzed by the Cox proportional hazards model [43]. Kaplan–Meier survival curves of mites for each treatment group were generated and survival was compared using the log-rank test. Additionally, the time to 50% mortality (LT_50_) was derived from the survival analysis. Alternatively, the median lethal concentration (LC_50_) at a particular time point of observation was calculated using a generalized linear model with binomially distributed error term.

The two-sided nonparametric Mann–Whitney test was used to compare the proportional reduction of mite counts and clinical score between the control group and the treatment group *in vivo* at Week 9 and for period Week 9–12. Missing values in the control group (due to salvage treatment) were replaced by the last observation carried forward. All analyses were performed in R v.3.5.1.

## Results

### Contact assays

**Fig. 1:**
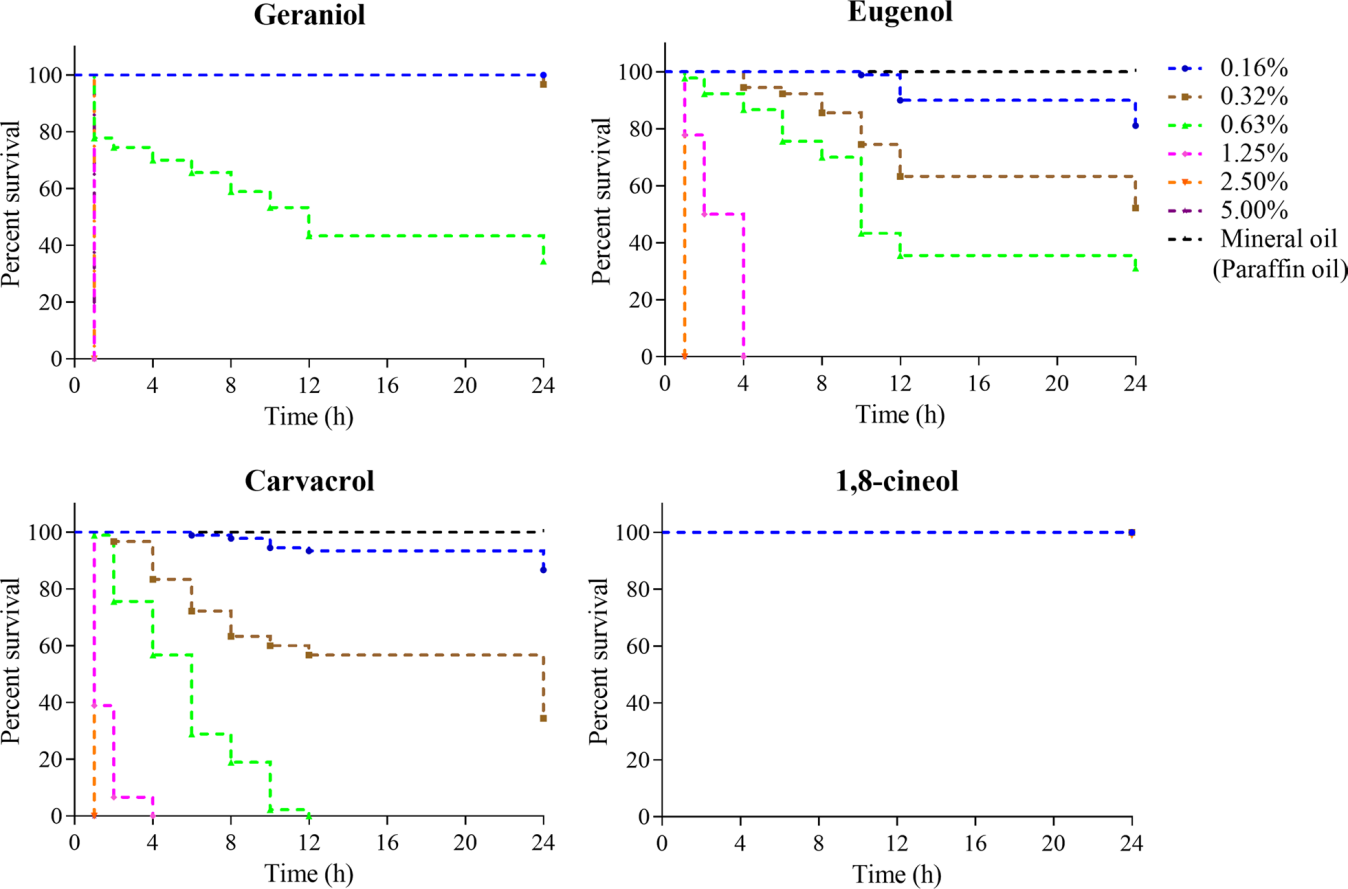
Survival curves of adult *P. ovis* mites exposed to different concentrations of geraniol, eugenol, carvacrol and 1,8-cineol in an acaricidal contact assay (immersion test). Eugenol was diluted with mineral oil, and the others were diluted with paraffin oil

After immersion with any of the four compounds, the mites displayed excited behavior and moved in circles prior to slowing down and eventually dying. Geraniol, eugenol and carvacrol displayed strong acaricidal activity against adult *P. ovis* mites *in vitro* upon direct contact with these compounds (Fig. 1). Indeed, when the concentration of these compounds exceeded 2.5% the mortality rate amongst adult mites was 100% within 1 h. At lower concentrations, eugenol and carvacrol showed a dose-dependent acaricidal activity and a low mortality rate was observed when adult mites were brought into contact for 24 h with a concentration of 0.16%. Geraniol had no activity at concentrations below 0.63% and 1,8-cineol did not show any acaricidal activity at any of the concentrations tested. Paraffin and mineral oil (negative controls) displayed no acaricidal activity against adult *P. ovis* (Fig. 1).

**Fig. 2:**
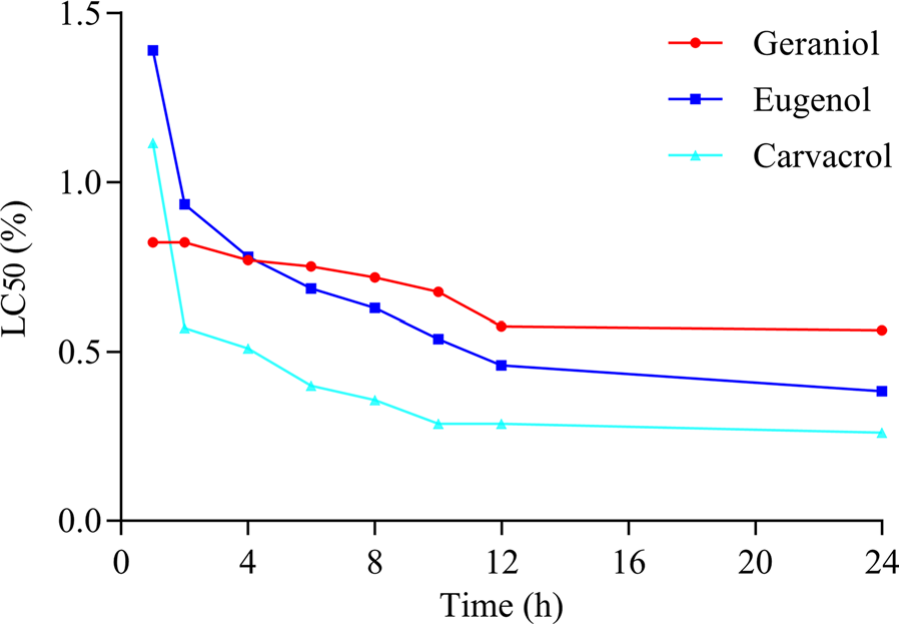
Estimated LC _50_ values of geraniol, eugenol, and carvacrol in an acaricidal contact assay (immersion test) as a function of time

Within 1 h of direct contact, geraniol displayed the lowest LC_50_ value compared with the other three compounds (Fig. 2). After 24 h of immersion, carvacrol displayed the lowest LC_50_ value (0.26%), followed by eugenol (0.38%) and geraniol (0.56%).

### Fumigation assays

**Fig. 3:**
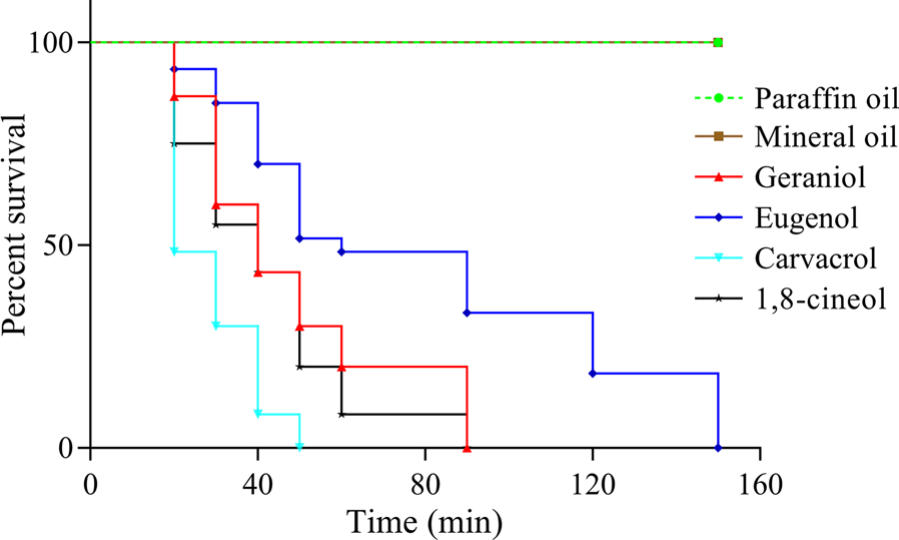
Survival curve of adult *Psoroptes ovis* mites exposed to undiluted geraniol, eugenol, carvacrol and 1,8-cineol in a fumigation assay

The LT_50_ values of geraniol, eugenol, carvacrol and 1,8-cineol in a fumigation assay (Fig. 3) were 40, 67, 24 and 35 min, respectively. Paraffin oil and mineral oil (control group) did not show any activity against adult mites. Paraffin oil and mineral oil (control group) did not show any activity against adult mites. All mites were killed within 50 min of treatment with carvacrol whereas geraniol, eugenol and 1,8-cineol needed 90, 150 and 90 min, respectively (Fig. 3).

### Residual assays

**Fig. 4:**
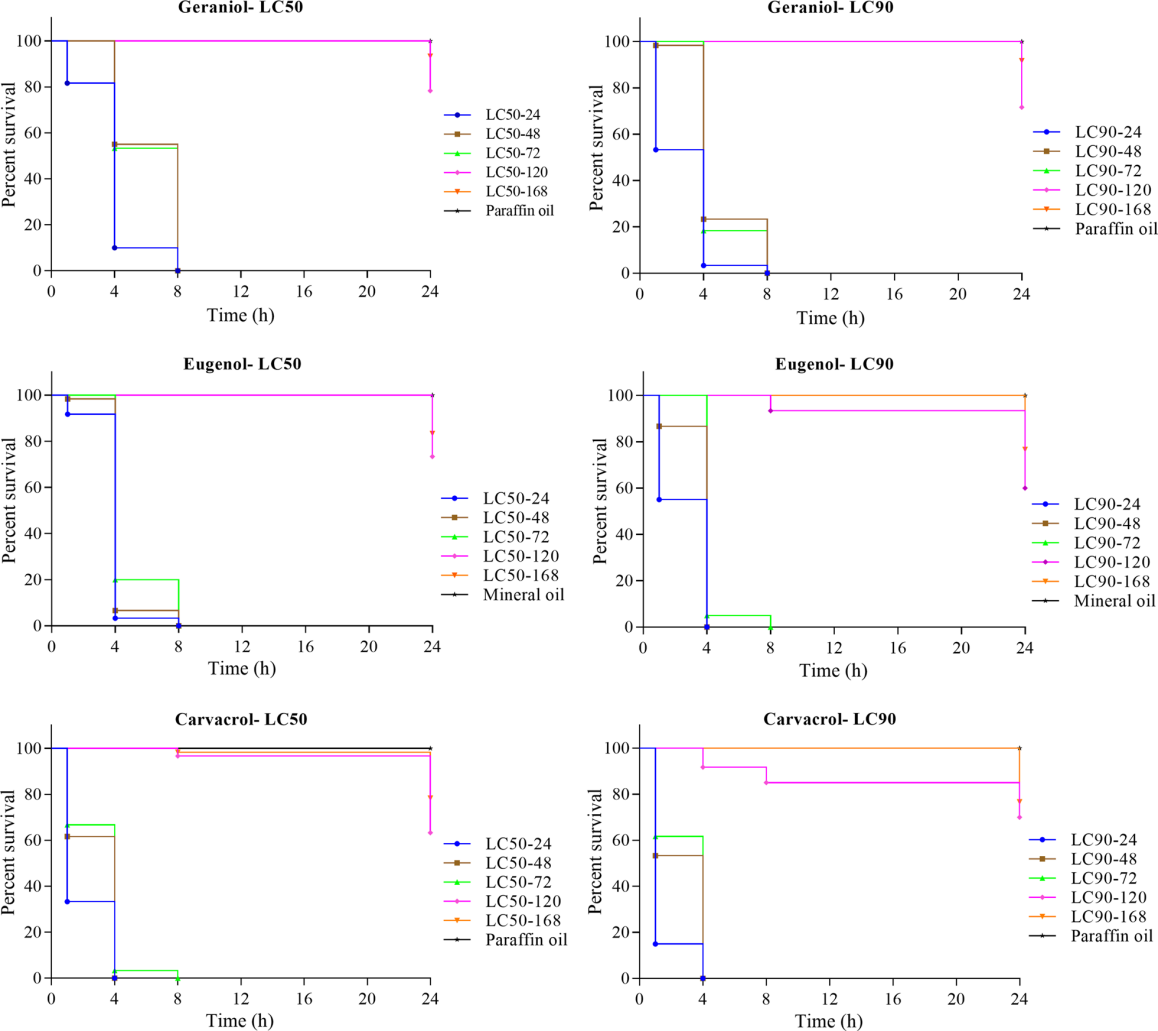
Survival curves of adult *P. ovis* mites exposed to LC_50_ and LC_90_ values of geraniol, eugenol, and carvacrol following incubation of these compounds for 24, 48, 72, 120 and 168 h in a residual activity assay

When carvacrol was incubated for 72 h at LC_90_ concentrations, it was still able to kill 100% of the mites following 4 h of immersion, while geraniol and eugenol incubated for 72 h at LC_90_, needed 8 h of immersion to kill all adult mites. When incubated for 72 h at LC_50_, geraniol, eugenol and carvacrol needed 8 h of immersion to kill all adult mites. Following 120 h of incubation the efficacy of all three natural compounds declined sharply (Fig. 4).

### *In vivo* assay

In the treated group, no living mites were recovered at the third treatment (Week 9). In contrast, the number of living mites in the control group was increased at Week 9, and the proportional reduction of mite counts was significantly higher in the treatment group as compared to the control group after the third treatment (*U* = 0, *Z* = − 2.88, *P* = 0.0028). In the post-treatment period (Week 9–12), a few mites were recovered from 1 or 2 animals in the treated group (Table 1). In the control group, the mite population increased after sham treatment, resulting in a significant difference (*U* = 0, *Z* = − 2.88, *P* = 0.0043) between the mean proportional reduction of mite counts in both groups over the period Week 9–12.

**Table 1:** Mite counts of *P. ovis* infested calves treated with carvacrol and Tween-80 (treatment group) or Tween-80 alone (control group), before infestation (W0), before treatment (W6) and throughout the post-treatment period (W9–W12)

Animal number		Mite counts (weeks after infection)					
		W0	W6	W9	W10	W11	W12
Treatment	7530	0	40	0	0	2	2
	2517	0	49	0	1	1	2
	992	0	10	0	0	0	0
	828	0	13	0	0	0	0
	8386	0	385	0	0	0	0
	9647	0	184	0	0	0	0
	Mean	0	113.5	0	0.2	0.5	0.7
Control	7937	0	669	486	634	635	–
	9392	0	59	258	446	214	–
	8670	0	346	497	360	419	78
	2141	0	18	265	561	422	384
	1345	0	463	618	704	–	–
	2138	0	140	432	572	–	–
	Mean	0	282.5	426	546.2		

*Note*: “–” The control group was treated topically with amitraz (Taktic^®^) at the recommended dose

**Fig. 5:**
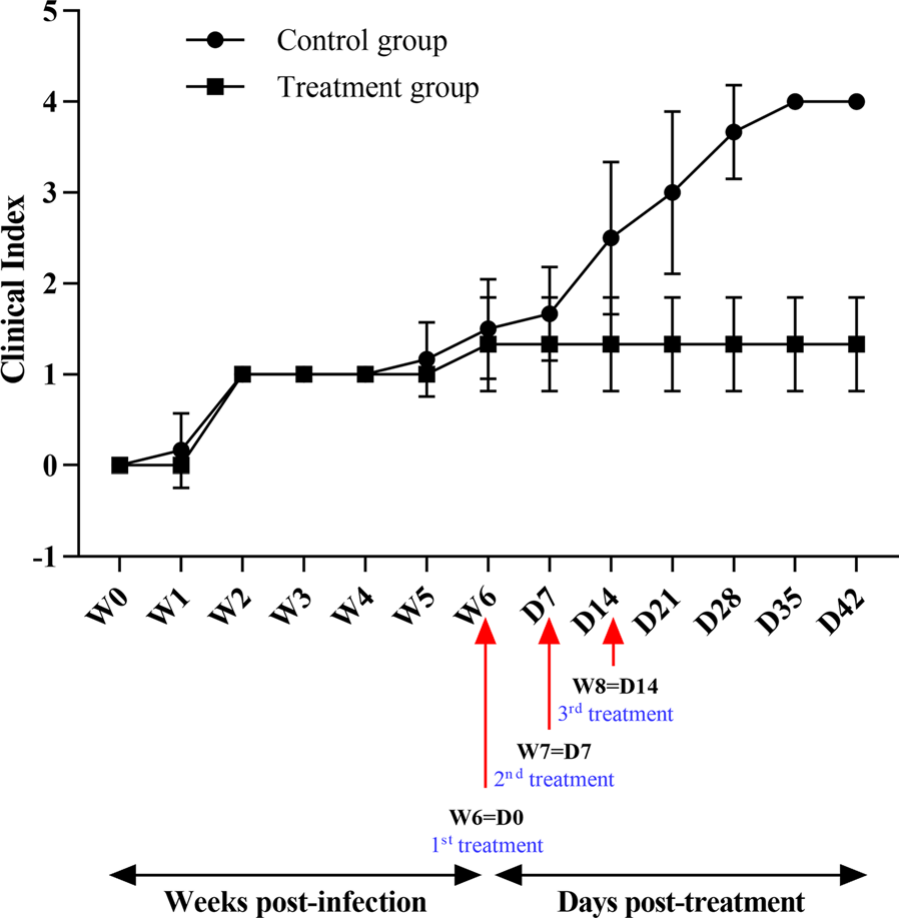
Clinical index (mean ± SD) of *P. ovis* infested calves treated with carvacrol and Tween-80, before infestation (Week 0), 6 weeks after infestation, prior to treatment (W6 = D0) and throughout the post-treatment period. Clinical index is based on the lesion-involved skin areas (0, healthy skin; 1, lesions surface < 10% of body surface; 2, lesions surface 10–20% of body surface; 3, lesions surface 20–30% of body surface; 4, lesions surface > 30% of body surface)

Active lesions with wet crusts adherent to the skin were observed on the withers and back of all calves after 6 weeks of infestation. In the control group, the CI increased during the whole study period. According to WAAVP guidelines [40], all control group animals were treated topically with amitraz at week 12 or before for animal welfare reasons. The CI of the control group was significantly higher than that of the carvacrol group after the third treatment (*U* = 0, *Z* = − 2.88, *P* = 0.0023) (Fig. 5). At the end of the animal trial (Week 9–12), a significant difference remained between control and treatment group (*U* = 0, *Z* = − 2.88, *P* = 0.0027). Although the surface of the skin lesions in the treatment group did not decrease after treatment, the lesions appearance changed from active to healing lesions from one week following treatment. Indeed, as the experiment developed, the treated calves appeared to suffer less from pruritus and self-trauma than the control group.

### Skin irritation assay

**Fig. 6:**
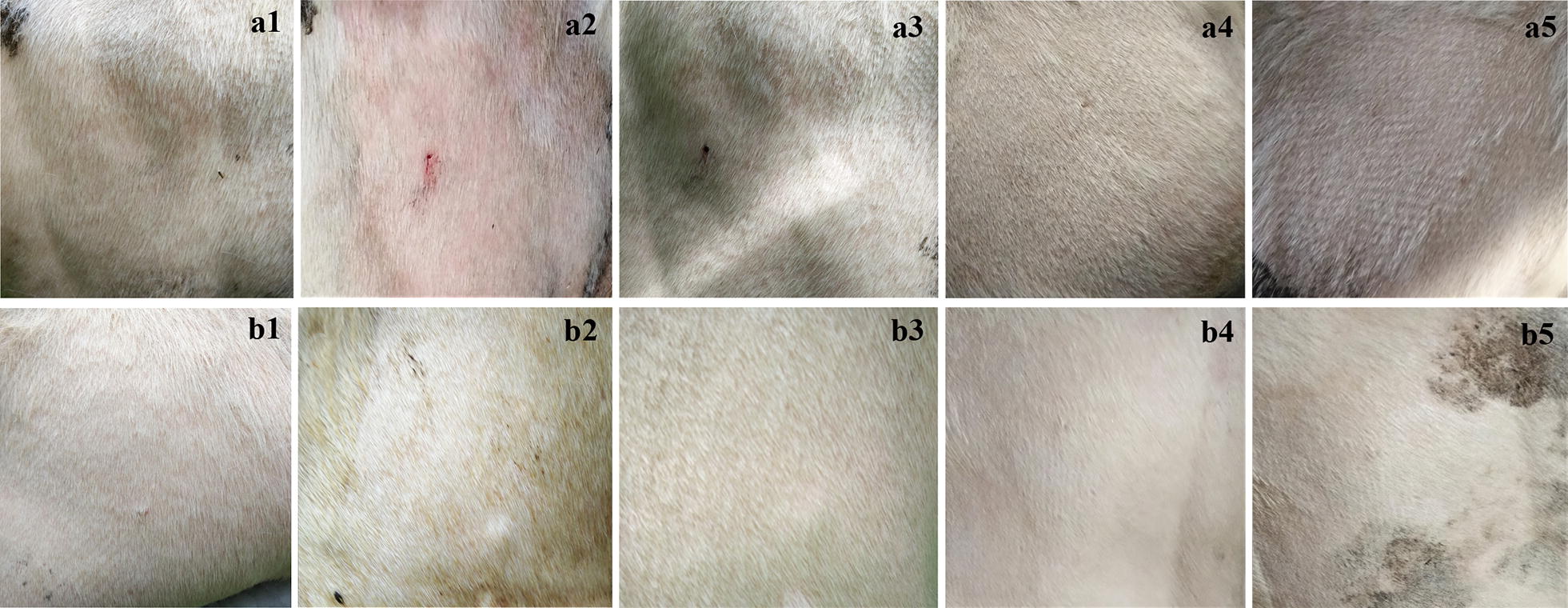
Clinical observations of the BB cattle skin treated with 2% carvacrol in Tween-80 (2%) (**a1**–**a5**) and only Tween-80 (2%) (**b1**–**b5**) at 0 min (**a1**, **b1**), 20 min (**a2**, **b2**), 6 h (**a3**, **b3**), 24 h (**a4**, **b4**) and 48 h (**a5**, **b5**)

Following the application of 2% carvacrol with Tween-80 (2%) to calves’ skin, the treated calves developed mild erythema 20 min post-treatment (Fig. 6). This redness had disappeared by 6 h post-treatment. There was no reaction in the control group. Oedema was not observed in any animal. The width of the *stratum corneum* and the numbers of eosinophils and mast cells in the superficial and deep dermis were not significantly different between the treatment group and the control group (results not shown).

## Discussion

Prior work has documented that chemical acaricidal products, such as ivermectin long-acting injectable, protected cattle against *P. ovis* for as long as 8 weeks [7, 9]. However, the macrocyclic lactones have long meat withdrawal periods, before which treated sheep or cattle cannot be slaughtered for human consumption [44]. Recent studies also reported resistance to macrocyclic lactones in *P. ovis* in sheep and cattle [11, 12]. To solve those issues, much effort has been focused on the development of natural acaricides, which may decrease the negative impact of synthetic acaricides, such as residues, resistance and environmental pollution. In this respect, plant-derived essential oil against ectoparasites may be effective, biodegradable and less harmful to the environment [31, 45].

Previous studies have shown that a large number of essential oils had acaricidal activity *in vitro* and *in vivo*. Clove oil, tea tree oil, eucalyptus oil, eupatorium extracts and lemon oil caused significant mortality in *S. scabiei* [14, 17, 23, 24, 33, 46]; cinnamon oil, oregano oil, Laurus and rhododendron in *P. cuniculi* [13, 18, 20, 25, 27]; and trans-cinnamic acid in *P. ovis* [15]. Although the acaricidal efficacy of many essential oils has been well documented, the involved mechanism and effective ingredients of the acaricidal activity are barely understood. This is due to the complex composition of essential oils. Further studies suggested that the efficacy of essential oils for control of ectoparasites can be attributed to the major compounds such as thymol, geraniol, eugenol, carvacrol and terpinen-4-ol [13, 17, 35, 38]. The present study aimed to select potential pure compounds against *P. ovis in vitro* and *in vivo*.

In the present study, geraniol, eugenol and carvacrol exhibited a significant time- and concentration-dependent acaricidal activity against *P. ovis*. Contact assays demonstrated that carvacrol at concentrations higher than 0.63% (equal to 6.15 mg/ml) was able to kill all mites after 10 h; whereas, at concentrations lower than 0.32% (equal to 3.07 mg/ml), the efficacy of carvacrol was limited (Fig. 1). The results are in line with a previous report, where 1% carvacrol led to 100% mortality of *P. ovis* mites and 0.1% carvacrol showed limited effect on the mites 72 h post-treatment [38]. However, Shang et al. [36] found that the LC_50_ of carvacrol at 24 h post-treatment against *P. cuniculi* was only 336.51 μg/ml, suggesting a higher efficacy of carvacrol.

Our study demonstrated that the minimum concentration of eugenol leading to 100% mortality is 1.25% (equal to 13.34 mg/ml) after 24 h (Fig. 1), and the LC_50_ of eugenol at 24 h was 0.38% (equal to 4.05 mg/ml) (Fig. 2). Similarly, Perrucci et al. [35] showed that eugenol at concentrations more than 0.125% killed nearly 100% of *P. cuniculi* mites after direct contact for 48 h. However, in the study of Ma et al. [37], concentrations of eugenol ≥ 2 mg/ml killed all *P. cuniculi* mites in less than 18 h. In addition, Shang et al. [36] reported that the LC_50_ of eugenol against *P. cuniculi* mites at 24 h post treatment was only 56.61 μg/ml. These results were markedly different with our results, as Ma et al. [37] and Shang et al. [36] reported much lower effective concentrations for carvacrol and/or eugenol than in the present study. Considering that *P. cuniculi* and *P. ovis* are the same species [47], this inconsistency could be attributed to differences in the experimental design, such as the use of filter paper *versus* immersion and the use of different solvents. For instance, during the incubation of mites on filter paper in the study of Shang [36], the solvent (mainly water) could have evaporated, resulting in increasing concentrations of the active compound and a higher efficacy. In contrast, eugenol does not easily evaporate from mineral oil.

In the present study, geraniol, at concentrations higher than 1.25%, showed 100% acaricidal activity after one hour of contact with *P. ovis* mites. This mortality decreased significantly to 40% when geraniol was diluted to 0.63% (equal to 5.54 mg/ml) and the mortality was 0% at concentrations lower than 0.63% (Fig. 1). In the study of Dunn et al. [38], 1% geraniol induced 100% mortality of *P. ovis* after 72 h, but 0.1% geraniol showed limited effect on the mites, which is in line with our results. Traina et al. [48] showed that the concentrations of geraniol above 5% were able to kill all mites [*Otodectes cynotis* (Acari: Psoroptidae)] within one hour, while it took 14 h for 1% geraniol to reach the same mortality. Exposure of *D. gallinae* to 0.5–2% of geraniol over a period of 24 h resulted in 100% mortality at all concentrations used [49]. These results indicate that geraniol at a high concentration shows a rapid acaricidal effect against a wide range of ectoparasites, but at low concentrations the efficacy may vary between mite species.

1,8-Cineol had no acaricidal activity on *P. ovis* 24 h post-treatment in our study, even at a concentration of 5%. Interestingly, Hu et al. [34] showed by immersion assays that when the concentration of 1,8-cineol was lower than 5%, 1,8-cineol had efficacy against *S. scabiei* larvae, suggesting that a higher concentration of 1,8-cineol is needed to kill adult *P. ovis* mites than *S. scabiei* larvae.

The results of the fumigation assays demonstrated a strong efficacy of the tested natural compounds on *P. ovis* (Fig. 3). However, the fumigant efficacy of the four compounds did not exactly match their contact efficacy. For example, 1,8-cineole had strong toxicity by fumigation, despite low contact activity. Similar differences were reported in studies on contact and fumigant effects of essential oils against *Sitophilus oryzae* [50], *Tribolium castaneum* [51] and *S. scabiei* [14]. Those results showed that eucalyptus, rosemary and tea tree oil, which contain 1,8-cineole, were more active by fumigation than by contact. The fumigant efficacy of essential oils appears to be linked to the monoterpenes to which the mites were exposed. Perrucci et al. [35] showed that geraniol and eugenol had potent acaricidal activity in inhalation assays against *P. cuniculi*, whereas the efficacy of linalyl acetate and estragole were not good. The difference between contact and fumigation bioassays could also be due to ingestion of the compounds in the contact assays.

The residual efficacy of the tested compounds decreased sharply after 120 h exposure (Fig. 4). This may be due to the evaporation of the natural compounds, resulting in a decreased mortality of mites with time. Hence, the present formulation as an effective control agent for *P. ovis* depends both on its use in relatively high concentrations and short treatment intervals, as the acaricidal activity quickly fades.

As yet, no *in vivo* tests of the acaricidal efficacy of carvacrol against *P. ovis* in cattle have been published. After three topical treatments, the proportional reduction in mite counts was significantly higher in the treatment group, compared to the control group (Table 1). Although the lesion size did not decrease after treatment, the appearance of the lesions changed from active to healing lesions (Fig. 5). On two calves in the treatment group, two live mites were observed until the end of this experiment. Survival of even a few mites may pose a risk for recolonization of the body surface after treatment. Re-emergence of psoroptic mange after apparent healing of treated cattle is common [52]. Wall et al. [15] also showed that application of *trans*-cinnamic acid (10%) by spraying the active mange lesions in sheep, resulted in only 87.5% reduction of *P. ovis* mite counts. This indicates that a full protection against *P. ovis* in sheep and cattle may require treatment of a larger area (e.g. the whole body) or more frequent treatments. In contrast, lemon oil (20%), neem oil (25 %) and oregano oil (5%) were able to cause 100% mortality of *S. scabiei* and *P. cuniculi* in rabbits [13, 19, 23]. This high efficacy in rabbits may be due to a higher susceptibility of *S. scabiei* to essential oils, which was also seen *in vitro* [34], or to the restricted habitat of *P. cuniculi* in the ear canal, which facilitates treatment. Moreover, these oils (lemon, neem and oregano) were also used at much higher concentrations than in the present study and this may have increased the effect.

Topical application of essential oils can induce concentration dependent skin irritation or inflammation [53]. It is reported that allergy and skin irritation caused by tea tree oil can be significantly reduced by diluting the tea tree oil [33, 54–56]. In the present study, only slight and transient erythema was observed on the treated calves’ skin after 20 min (Fig. 6). Furthermore, the number of eosinophils and mast cells in this study were not significantly different between the treatment group and control group (results not shown). Klecak et al. [57] showed that the minimum dermal sensitizing concentration of carvacrol was 3% on guinea pigs’ skin. Alternatively, rat skin was used for an *in vitro* test to evaluate the corrosivity of carvacrol (concentration not stated) for 2 and 24 h, showing no corrosive activity [58]. In addition, the *in vivo* genotoxicity of carvacrol was tested in rats, which suggested that carvacrol (81–810 mg/kg body weight) did not induce genotoxicity or oxidative DNA damage in any of tissues investigated [59]. It was presumed that 2% carvacrol could be used as a safe antiparasitic drug in protecting animals.

## Conclusions

In the present study, we demonstrated significant acaricidal activity against adult *P. ovis* mites of geraniol, eugenol and carvacrol using contact, fumigation and residual assays *in vitro*, and of 1,8-ceniol using a fumigation assay*. In vivo* evaluation of carvacrol in cattle resulted in 98.5% elimination of mites. In addition, topical treatment of calves with carvacrol only caused mild and transient local side effects. Taken together, these data indicated the potential of carvacrol as an acaricidal agent in the treatment of *P. ovis* infestations in cattle. The low toxicity and high biological activity of carvacrol make it a promising compound for the development of novel medicines, not only for cattle but potentially also for other animals and humans.

## Data Availability

All data generated or analysed during the present study are included in this published article.

## Abbreviations

LC50: median lethal concentration
LC90: 90% lethal concentration
MC: mite counts
CI: clinical index
LT50: median lethal time
